# Perioperative Anesthetic Considerations in HMG-CoA Lyase Deficiency: Case Report and Literature Review

**DOI:** 10.3390/jcm14207332

**Published:** 2025-10-17

**Authors:** Vasileia Nyktari, Georgios Papastratigakis, Alexandra Koulousi, Chrysi Mandola, Foteini Chaniotaki, Ioannis Goniotakis, Stavroula Ilia, Alexandra Papaioannou

**Affiliations:** 1 School of Medicine, University of Crete, 71500 Heraklion, Crete, Greece; papastratigakisg@gmail.com (G.P.); stavroula.ilia@uoc.gr (S.I.); papaioaa@uoc.gr (A.P.); 2 Anesthesiology Department, University General Hospital of Heraklion, 71110 Heraklion, Crete, Greece; akoulousi@gmail.com (A.K.); mandolachrysa24@gmail.com (C.M.); fchaniotaki99@gmail.com (F.C.); 3 Pediatric Intensive Care Unit, University General Hospital of Heraklion, 71110 Heraklion, Crete, Greece; ioannisgoniotakis@gmail.com

**Keywords:** case reports, 3-hydroxy-3-methylglutaryl CoA lyase deficiency, fatty acid oxidation disorders, anesthesia, general, perioperative care, adolescent, emergency surgical procedures

## Abstract

**Background/Objectives**: 3-Hydroxy-3-methylglutaryl-CoA lyase deficiency (HMGCLD) is an extremely rare autosomal recessive metabolic disorder caused by mutations in the HMGCL gene. HMGCLD disrupts ketogenesis and β-oxidation, leading to energy failure during fasting or stress, with clinical episodes characterized by hypoglycemia, hyperammonemia, lactic acidosis, and encephalopathy. Only 211 cases have been reported worldwide, with no prior reports on anesthetic management in these patients. **Methods**: We report a 14.5-year-old girl with known HMGCLD who was admitted with abdominal pain and nausea following a fatty meal. Imaging confirmed acute cholecystitis. Initial conservative management failed due to persistent vomiting and inability to tolerate feeding. Deviation from the metabolic protocol led to lactic acidosis and hypoglycemia, requiring intensive care with bicarbonate, carnitine, and glucose infusion. Once optimized, she underwent emergency laparoscopic cholecystectomy under sevoflurane-based anesthesia. Propofol was avoided, given the patient’s compromised lipid metabolism. Intraoperative glucose and acid-base status were closely monitored, with balanced dextrose-based fluids. **Results**: The patient remained hemodynamically stable throughout and was discharged three days postoperatively. **Conclusions**: This case highlights the anesthetic challenges of HMGCLD, where system-level miscommunication can trigger severe metabolic decompensation. A review of the literature emphasizes fasting avoidance, continuous glucose supplementation, careful drug and fluid selection, and multidisciplinary coordination. This report provides the first anesthetic roadmap for HMGCLD, underscoring the need for individualized care and meticulous perioperative metabolic control.

## 1. Introduction

3-Hydroxy-3-methylglutaryl-CoA (HMG-CoA) lyase deficiency (HMGCLD) is an autosomal recessive disorder first described by Faull et al. [1]. It results from mutations in the HMGCL gene [2,3], disrupting pathways of ketogenesis and leucine catabolism. Considered extremely rare, its estimated prevalence is less than 1 in 100,000 live births, with approximately 200 cases reported in the literature [4,5].

Routine newborn screening programs generally do not include HMGCLD. In the majority of patients, diagnosis occurs after the first episode of hypoketotic hypoglycemia or metabolic acidosis in infancy. Typical biochemical findings include the accumulation of leucine metabolites during urinary organic acid analysis. Molecular confirmation is achieved by identifying pathogenic variants in the HMGCL gene. Early recognition, particularly in siblings of affected individuals, allows timely management and prevention of severe metabolic decompensation [3,4]. HMG-CoA lyase deficiency is an autosomal recessive disorder [1,5]; therefore, genetic counseling is important to inform families about recurrence risk and reproductive options.

Clinically, it manifests through recurrent episodes of metabolic acidosis, hyperammonemia, and hypoglycemia [2,6]. During prolonged fasting, glycogen stores become depleted, leading to lactic acidosis due to impaired β-oxidation and an inability to produce ketones from fatty acids [1].

HMGCLD is a mitochondrial enzyme disorder essential for ketogenesis and amino acid metabolism. Metabolic disorders like this increase anesthesia-related risks, including metabolic decompensation, drug interactions, organ dysfunction, and physiological stresses that anesthesia can worsen. To our knowledge, there are no published case reports regarding the anesthetic management of patients with HMGCLD. This report provides the first documented approach to perioperative anesthetic management in this population, offering practical guidance to avoid life-threatening decompensation in similar cases. We present a pediatric patient with HMGCLD who underwent emergency surgery and review the literature on perioperative care in fatty acid oxidation disorders. The objective of this article is to illustrate the anesthetic considerations in such patients and to underscore the critical importance of individualized anesthetic planning and strict adherence to metabolic protocols. Written informed consent for publication was obtained from the patient’s guardians.

## 2. Case Presentation

### 2.1. Pre-Hospital Phase and Hospital Admission to Pediatric Department

A 14.5-year-old girl (72 kg, 160 cm) with a known history of HMG-CoA lyase deficiency (HMGCLD) was admitted to our Pediatric Department for evaluation of abdominal pain and nausea following the ingestion of a fatty meal. An initial abdominal ultrasound suggested cholelithiasis. She was managed conservatively with intravenous glucose-containing fluids and was able to tolerate a fat-free diet.

### 2.2. Hospital Phase—Deterioration and Admission to Surgery Department

Despite conservative management, her condition worsened over three days, prompting reevaluation, which confirmed acute cholecystitis. She was transferred to the General Surgery Department for further treatment, including an NPO (nil per os) protocol, symptomatic therapy, and intravenous Maintelyte solution. During this period, she developed recurrent moderate to severe hypoglycemia, requiring glucose boluses. After two days of marginal clinical improvement, a fluid diet was gradually introduced, but she experienced recurrent vomiting with each feeding over the subsequent five days.

On day 10, her IV fluids were switched to Ringer’s Lactate, which resulted in clinical and laboratory deterioration within 12 h, including worsening acidosis and hypoglycemia. Persistent vomiting prompted reevaluation, and surgical intervention was decided, following consultation with anesthesiology and intensive care specialists.

### 2.3. Admission to PICU and Preoperative Optimization

Due to ongoing lactic acidosis and hypoglycemia, she was transferred to the Pediatric Intensive Care Unit (PICU) for stabilization prior to surgery. In the PICU, an arterial line was placed, and she received bicarbonate, L-carnitine, and 10% dextrose with electrolytes (NaCl, KCl, MgSO_4_) at 150 mL/h, leading to significant clinical and biochemical improvement.

### 2.4. Intraoperative Management

Following stabilization, she underwent emergency laparoscopic cholecystectomy under standard ASA monitoring and invasive blood pressure measurement. Anesthesia was induced intravenously with midazolam (5 mg), fentanyl (250 mcg), and cisatracurium (15 mg), and supplemented with sevoflurane at 6% briefly for the induction stage with uneventful intubation using a size 7 cuffed endotracheal tube. Anesthesia was maintained with sevoflurane at a MAC of 1.0. Ventilation was set to FiO_2_ 35%, employing decelerating flow and volume guarantee. Continuous monitoring of arterial blood gases and glucose levels was conducted to ensure metabolic stability, as summarized in Table 1.

The patient received maintenance fluids of 0.9% normal saline at 200 mL/h, supplemented with an enriched glucose solution at 120 mL/h. Antiemetic prophylaxis included dexamethasone (8 mg) and ondansetron (4 mg). Postoperative analgesia was ensured with a multimodal analgesic protocol, including a 30 min infusion of dexmedetomidine (25 μg), a 3 mg intravenous bolus of morphine, and a preoperative intravenous dose of paracetamol (1 g). The procedure lasted approximately 40 min and was uneventful, with stable hemodynamics throughout. Neuromuscular blockade was reversed with 2.5 mg of neostigmine and 1 mg of atropine, and the patient recovered smoothly.

### 2.5. Postoperative Course

The patient remained in the PICU for two days for close monitoring, followed by one additional day in the surgical ward. Her postoperative course was uncomplicated, characterized by minimal pain and a high level of patient satisfaction.

## 3. Discussion

**Figure 1:**
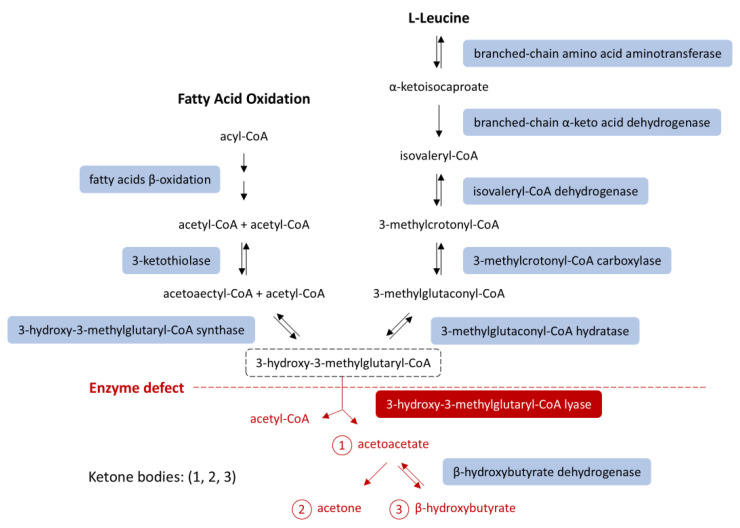
Ketone body synthesis from fatty acid β-oxidation and leucine catabolism. HMG-CoA lyase deficiency (red) blocks conversion of HMG-CoA into acetoacetate, impairing ketogenesis. An HMG-CoA lyase deficiency (red) impairs ketogenesis [8,9]. Modified from [8], licensed under CC BY 3.0 (https://creativecommons.org/licenses/by/3.0/ (accessed on 14 October 2025)). Abbreviations: HMG-CoA, 3-hydroxy-3-methylglutaryl-coenzyme A.

The HMGCL gene encodes the enzyme 3-hydroxy-3-methylglutaryl-CoA lyase (3-HMG-CoA lyase), which serves as the final mitochondrial enzyme in ketone production pathways, specifically in fatty acid oxidation and leucine catabolism [2,5] (Figure 1). The deficiency of this enzyme prevents the production of acetoacetate and acetyl-CoA from these pathways, leading to an energy deficit during starvation and causing profound hypoglycemia [5,6,7]. This explains why patients with HMGCL deficiency are particularly vulnerable during fasting or prolonged NPO status, as their ability to generate ketone bodies, the key alternative energy sources during fasting, is impaired [2,6,7].

In a healthy individual, periods of starvation trigger hypoglycemia, which, in turn, activates compensatory metabolic pathways. Specifically, increased glucagon production stimulates hepatic glycogenolysis, as well as hepatic and renal gluconeogenesis [7,10]. When glucose is unavailable, glucogenic amino acids, such as alanine and aspartate, serve as gluconeogenic substrates [11]. As prolonged fasting depletes glycogen stores, lipolysis becomes an important energy source.

During lipolysis, each triacylglycerol molecule yields glycerol and free fatty acids. Glycerol enters the gluconeogenic pathway, while free fatty acids undergo beta-oxidation in the inner mitochondrial membrane, producing acetyl-CoA in the liver [12,13]. In addition to transporting fatty acids across mitochondrial membranes, carnitine aids in clearing toxic byproducts from the organelle [14]. Acetyl-CoA, derived from fatty acid oxidation, then enters the citric acid cycle or is converted into ketone bodies. Ketones are essential for glucose-dependent tissues like the brain and the heart [11,15]. enabling these tissues to produce energy during glucose deprivation [13].

Lipolysis and gluconeogenesis are intricately interconnected. Specifically, beta-oxidation is a major supplier for molecules such as NADH+ and acetyl-CoA, which in turn stimulate pyruvate carboxylase (PC) and inhibit pyruvate dehydrogenase (PDH) enzymes [16]. PC is the enzyme responsible for the conversion of pyruvate to oxaloacetate, to serve as a substrate for gluconeogenesis. PDH, on the other hand, converts pyruvate into acetyl-CoA, thus promoting glycolysis when fatty acid oxidation is impaired. Gluconeogenic substrates such as alanine and lactate are normally converted into pyruvate to enter the gluconeogenesis pathway. As a result, increased lipolysis and fatty acid oxidation promote diversion of pyruvate towards gluconeogenesis and inhibit glycolysis in the liver, preserving the available glucose for other tissues [15,16]. Through this delicate balance, there is an ongoing supply of glucose and ketone bodies to the tissues.

**Figure 2:**
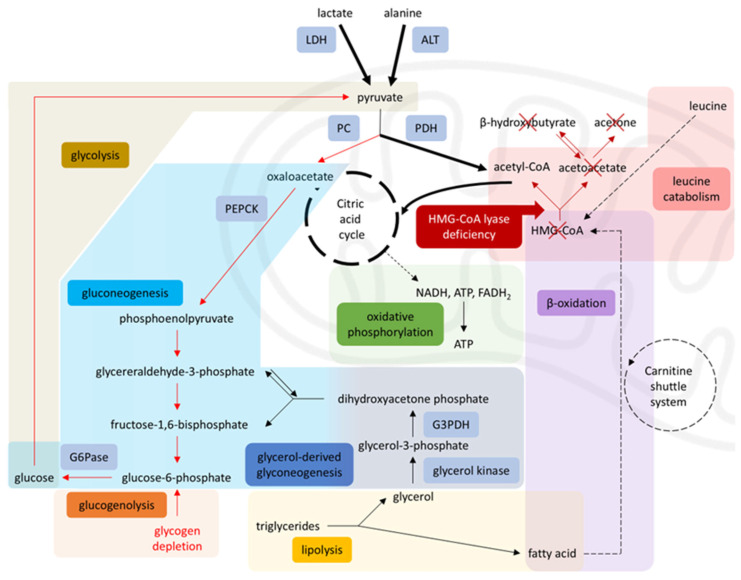
Disrupted metabolism in HMG-CoA lyase deficiency. Impaired β-oxidation and leucine catabolism prevent acetyl-CoA production, inhibiting gluconeogenesis (via PC) and enhancing glycolysis (via PDH), leading to energy failure during starvation, diverting pyruvate toward the citric acid cycle and limiting its availability for gluconeogenesis. Solid arrows = single-step processes; dashed arrows = simplified multi-step processes; Red arrows = reduced activity; Red cross = blocked pathways; Bold arrows = increased activity. A stylized mitochondrion highlights the intra-mitochondrial pathways. Modified from [17], licensed under CC BY 3.0 (https://creativecommons.org/licenses/by/3.0/). Abbreviations: LDH: lactate dehydrogenase, ALT: alanine aminotransferase, PC: pyruvate carboxylase, PDH: pyruvate dehydrogenase, PEPCK: phosphoenolpyruvate carboxykinase, G6Pase: glucose-6-phosphatase, G3PDH: glyceraldehyde-3-phosphate dehydrogenase, HMG-CoA: 3-hydroxy-3-methylglutaryl coenzyme A, NADH: nicotinamide-adenine dinucleotide, FADH_2_: flavin adenine dinucleotide [4,8,9,17].

However, in HMGCLD, as seen in our patient, these key metabolic processes are disrupted. Due to impaired beta-oxidation, the liver cannot produce ketones or effectively utilize gluconeogenic substrates like lactate and alanine. Additionally, 3-HMG-CoA lyase plays a role in leucine catabolism into acetoacetate and acetyl-CoA (Figure 1) [1,4]. During starvation, when glucose intake is absent and lipolysis is insufficient for energy demands, the body relies on protein catabolism and gluconeogenesis. While proteolysis occurs, leucine cannot be used for energy production because it cannot be converted into ketone bodies [2,3]. Consequently, other ketogenic and glucogenic amino acids enter a compromised gluconeogenic cycle [16] (Figure 2).

This metabolic rigidity means that even brief catabolic stressors, such as prolonged fasting, vaccination, infection or surgical stress, can precipitate metabolic crisis. in patients with HMG-CoA lyase or synthase deficiency [3,15,16]. As shown by Thompson et al. [10], even short fasting periods may cause severe hypoketotic hypoglycemia, with clinical manifestations ranging from vomiting, food refusal, and altered mental status to encephalopathy, seizures, coma, or even death. This highlights the crucial role of hepatic ketogenesis in maintaining cerebral energy balance during catabolic stress.

In our patient, a week-long fast, complicated by emesis upon feeding attempts, had not yet resulted in metabolic decompensation due to continuous intravenous supplementation with exogenous glucose, initiated at 8 mg/kg/min and titrated based on serial glucose monitoring to maintain euglycemia. However, following a transition to Ringer’s Lactate, a change implemented without immediate glucose replacement, the patient was effectively deprived of glucose supplementation, precipitating clinical deterioration. As evidenced in Table 1, serum glucose concentrations declined to 74 mg/dL pre-PICU within 6 h, necessitating prompt intervention.

Laboratory findings during episodes of decompensation typically reveal profound hypoglycemia (without ketosis), hyperammonemia, lactic acidosis, and abnormal liver function tests—all of which were present in our case. Common anticipated complications included hepatomegaly, cardiomyopathy, arrhythmias, skeletal myopathy, rhabdomyolysis, pancreatitis due to lipotoxicity, and energy deficiency [3,15].

Our patient was diagnosed with HMG-CoA lyase deficiency (HMGCLD) shortly after birth. Home management involved adequate caloric intake, frequent meals, and moderate restriction of protein and fat, as recommended in the literature [5,18]. She was not on regular L-carnitine supplementation due to the lack of consensus in the literature [9,19]. This was the second episode of metabolic decompensation requiring pediatric intensive care unit (PICU) admission, out of a total of 25 mild decompensation episodes during our patient’s lifetime. Metabolic decompensation is particularly critical, as approximately one in three patients may suffer permanent, severe neurological deficits [3].

Clinical awareness and perioperative protocols should emphasize strict fasting avoidance, early administration of glucose either orally or intravenously, and close metabolic monitoring to prevent life-threatening complications. In cases of severe metabolic acidosis (HCO_3_^−^ < 16 mEq/L), the administration of intravenous bicarbonate at a dose of 1 mEq/kg is recommended [4,18]. In our case, the patient’s HCO_3_^−^ levels upon admission to the PICU were very low (15 mEq/L), and she, therefore, received an IV infusion of 70 mEq NaHCO_3_ over 2 h.

Some authors suggest intravenous L-carnitine administration at 30–50 mg/kg/day, which was also given during preoperative optimization to prevent secondary L-carnitine deficiency, oxidative stress, and intracellular depletion of free coenzyme A. Carnitine molecules typically bind to the toxic products that accumulate due to impaired fatty acid metabolism, facilitating their excretion via urine. Secondary L-carnitine deficiency may impair the neutralization of oxygen free radicals and lead to the buildup of lipotoxic byproducts [20].

It is now evident that patients with HMGCLD require very careful anesthetic planning. Since there are no reported cases specifically detailing anesthetic management for these patients, we based our approach on published case reports of individuals with other beta-oxidation deficiencies [21,22,23] and general practice guidelines [4]. The selection of anesthetic induction and maintenance agents for our patient with HMG-CoA lyase deficiency was guided by principles of minimizing disruption to mitochondrial function and preserving metabolic stability.

Midazolam (5 mg) was selected for its anxiolytic and sedative properties, coupled with a comparatively benign cardiovascular profile. This relatively low dose was administered to achieve adequate sedation whilst supplementing the inhalational anesthetic, thereby minimizing physiological stress in a metabolically vulnerable patient [24]. Fentanyl (250 mcg) was chosen to provide analgesia, based on evidence suggesting a relatively limited impact on mitochondrial respiration compared to alternative opioid analgesics. Dosing was carefully considered to balance effective analgesia with minimizing the potential for opioid-induced metabolic disturbances [25].

For maintenance of anesthesia, sevoflurane was deemed appropriate, given its capacity for rapid pulmonary elimination and the potential for prompt restoration of baseline mitochondrial function following cessation of the anesthetic [26]. The concentration for anesthesia maintenance was maintained at or below 1.0 MAC, carefully titrated to effect, to minimize the potential for excessive anesthetic depth and subsequent metabolic stress. Acknowledging the potential for Complex I inhibition associated with volatile halogenated agents, meticulous titration is suggested to mitigate potential risks [27]. Additionally, Hsieh et al. [28] highlights the potential for increased sensitivity to volatile anesthetics, specifically sevoflurane, in patients with mitochondrial dysfunction, and careful titration of anesthetic agents is recommended. Therefore, monitoring of anesthetic depth, preferably with processed electroencephalogram (EEG) monitoring, may be a valuable adjunct for guiding anesthetic administration. Although HMGCLD, as a metabolic disorder with downstream effects on mitochondrial function, does not directly target Complex I, which is particularly associated with anesthetic hypersensitivity, it suggests that anesthetic choices should also be carefully made in patients with HMGCLD. While sevoflurane was employed in this case, we underscore the importance of close monitoring of arterial blood gases and glucose levels as a means to detect early signs of metabolic instability or anesthetic overdose. The decision to use sevoflurane, potentially based on its rapid emergence profile, was coupled with a deliberate strategy to mitigate potential risks. Conversely, we deliberately avoided propofol, owing to its established interactions at multiple sites within the mitochondrial electron transport chain and its potential to inhibit acylcarnitine transferase, thereby exacerbating metabolic derangements inherent to HMG-CoA lyase deficiency [29,30]. We also chose not to administer intravenous lidocaine, as the main antidote for inadvertent local anesthetic toxicity is lipid emulsion, which is contraindicated in our patient.

Regarding muscle relaxation, cisatracurium was preferred over rocuronium due to its lower hepatic metabolism, as it is primarily degraded by plasma esterases, advantageous given the patient’s mildly elevated liver enzymes. Additionally, if rocuronium had been used, the reversal with sugammadex would have raised theoretical concerns, as sugammadex can bind steroid hormones such as cortisol in experimental settings, although clinical studies in humans have not demonstrated clinically significant adrenal suppression [31]. In HMGCLD, disruptions in beta-oxidation may indirectly affect cholesterol synthesis and homeostasis by altering acetyl-CoA availability or cellular pathways regulating cholesterol metabolism [32]. This cautious approach avoided potential metabolic interference and ensured safe neuromuscular blockade.

When considering the use of regional anesthesia, a more thoughtful approach is necessary. We opted not to use local anesthetics for our patient because of concerns regarding the contraindication of lipid emulsion rescue in the event of local anesthetic toxicity. However, regional or neuraxial techniques may provide potential benefits for carefully selected patients with HMGCLD. These techniques can help reduce surgical stress and may permit less strict preoperative fasting requirements compared to general anesthesia. If planned and executed meticulously, along with close monitoring of metabolic parameters, regional anesthesia could lead to improved outcomes. Nevertheless, the theoretical risks associated with the contraindication of lipid emulsion and the potential for unpredictable metabolic responses must be carefully considered, along with a patient-specific risk-benefit assessment.

Another emerging challenge in these patients is the ability to quantify intraoperative stress. This parameter is a potentially valuable, but understudied, aspect of anesthetic management, especially in patients with metabolic disorders like HMG-CoA lyase deficiency. Surgical stress triggers catabolic pathways, and, in HMG-CoA lyase deficiency, this can lead to rapid metabolic decompensation due to impaired ketogenesis and gluconeogenesis. While direct measurement of stress hormones is not always feasible in real-time, surrogate markers derived from advanced anesthetic monitoring might offer insights. For example, Hung et al. describe a case where the Surgical Pleth Index (SPI), a plethysmograph-derived parameter reflecting autonomic nervous system activity, correlated with surgical stress and subsequent euglycemic diabetic ketoacidosis in a patient with Type 2 DM on SGLT2 inhibitors [33]. Although further research is needed to validate its utility, particularly in the context of HMG-CoA lyase deficiency, continuous monitoring of stress parameters such as SPI, alongside traditional hemodynamic variables, deserves exploration as a means to guide anesthetic management, anticipate metabolic crises, and potentially optimize patient outcomes. This could involve tailoring anesthetic depth, adjusting fluid and glucose administration, and employing strategies to minimize nociceptive stimuli.

Finally, we had to be diligent regarding the choice of intravenous fluids. We did not administer Ringer’s Lactated solution, as it contains lactate that could further deteriorate existing lactic metabolic acidosis. Instead, we administered Normal Saline 0.9% combined with Dextrose 10% with added electrolytes to correct hypoglycemia and electrolyte imbalances. All perioperative considerations with suggestions for anesthetic plan modification are illustrated in Table 2.

A major challenge was the necessity for coordinated collaboration among healthcare professionals from multiple departments. Due to case rarity, clinical decisions diverged from standard protocols, underscoring the critical importance of non-technical skills, particularly communication, situational awareness, and teamwork, vital for minimizing errors [34].

A key miscommunication involved IV glucose as a core element of HMGCLD management. On the fourth day of hospitalization, the glucose-rich solution (Maintelyte) was replaced with Ringer’s Lactated solution, which was consistent with standard practice in the surgical department but not appropriate for this patient’s fragile metabolic state. This substitution resulted in deterioration, highlighting the risks of applying routine protocols in complex metabolic disorders. This scenario exemplifies three recurrent issues in multidisciplinary collaborations: a lack of comprehensive situational awareness among team members, ineffective closed-loop communication, and confirmation bias, where clinicians default to familiar procedures based on previous experience rather than customized patient needs [34]. Recognizing these pitfalls is essential for optimizing interdisciplinary teamwork, especially in rare metabolic disorders requiring precise, collaborative decision-making. To mitigate these risks, implementing standardized communication protocols, such as pre-operative multidisciplinary meetings and structured handoffs, is crucial [35]. These strategies ensure that all members of the care team are aware of the patient’s unique metabolic needs and are prepared to respond effectively to potential complications.

## 4. Conclusions

HMGCLD is an exceptionally rare metabolic disturbance of fatty acid oxidation, with complex pathophysiology and only a few hundred cases reported. The absence of previous reports on anesthetic management makes this case particularly valuable, as it highlights the interplay between metabolic fragility, anesthetic drug choice, and system-level communication. Our experience underscores that individualized planning and multidisciplinary cooperation are essential to maintain stability and avoid life-threatening decompensation. Future research should focus on developing and validating standardized protocols and checklists for the perioperative management of patients with HMGCLD and other rare metabolic disorders. These tools, coupled with robust interdisciplinary communication strategies, have the potential to significantly improve patient outcomes and reduce the risk of life-threatening decompensation.

Key anesthetic lessons from this case include:

- Strict avoidance of prolonged fasting with early and continuous glucose supplementation.
- Avoidance of propofol and lipid-based therapies, due to impaired fatty acid metabolism.
- Use of lactate-free intravenous solutions to prevent worsening acidosis.
- Selection of cisatracurium over rocuronium, reducing hepatic load and avoiding sugammadex concerns.
- Close interdisciplinary communication to prevent errors such as inappropriate fluid substitutions.

Together, these principles provide practical guidance for the safe perioperative management of patients with HMGCLD and may inform future development of tailored anesthetic protocols for rare fatty acid oxidation disorders.

## Figures and Tables

**Table 1:** Perioperative values of arterial blood gases and glucose.

ABGs	Reference Values	Pre-PICU	PICU Prior to Surgery	Intraoperative 1	Intraoperative 2	Postoperative
pH	7.35–7.45	7.577	7.472	7.406	7.368	7.414
PaO_2_ (mmHg)	can vary based on FiO_2_; usually maintained ≥80 mmHg in stable patients	234	166	201	204	188.7
FiO_2_	adjusted to maintain adequate oxygenation	0.5	~0.28	0.35	0.35	~0.28
PaCO_2_ (mmHg)	35–45	16.4	28.4	30.6	34	33
BE (mmol/L)	−2 to +2	−4.8	−2.5	−5.1	−5.4	−1.3
HCO_3_ (mmol/L)	22–26	15	20.3	18.8	19.3	20.4
Lactate (mmol/L)	0.5–2.2	3.38	1.64	1.8	1.8	1.5
Glucose (mg/dL)	70–120	74	173	144	177	204

**Table 2:** Anesthetic considerations and recommendations for patients with HMG-CoA lyase deficiency.

Consideration	Recommendation	Rationale
Fasting	Strict avoidance, early and continuous glucose supplementation	Prevents hypoglycemia and metabolic decompensation
Anesthetic Technique	Regional or neuraxial techniques should be carefully considered based on the risks and benefits	May provide less stringent pre-operative fasting and potentially reduce intraoperative stress Intralipid, the antidote in case of local anesthetic toxicity (LAST) is contraindicated.
General Anesthesia	Avoid propofol and lipid-based therapies	Impaired fatty acid metabolism
	Avoid/limit the use of intravenous lidocaine	Avoidance of LAST and possible use of intralipid
	Volatiles Pro: rapid pulmonary elimination and termination of any possible effect Con: Possible hypersensitivity to sevoflurane and other volatiles in mitochondrial metabolic disorders	monitoring of anesthetic depth, preferably with processed electroencephalogram (EEG) monitoring, may be a valuable adjunct for guiding anesthetic administration
	Cautious use of ketamine	Avoid high doses to prevent increased energy consumption
Intravenous Fluids	Use lactate-free solutions (e.g., Normal Saline 0.9%) with Dextrose 10%	Prevents worsening lactic acidosis
Neuromuscular Blockers	Prefer cisatracurium over rocuronium	Cisatracurium has lower hepatic metabolism Avoid theoretical sugammadex concerns
Multidisciplinary communication Deviation from standard fasting and fluid administration protocols	Close coordination among healthcare professionals	Minimizes errors in fluid and medication management, ensures tailored care for this rare metabolic disorder

## Data Availability

No new data were created or analyzed in this study. Data sharing is not applicable to this article.

## Abbreviations

The following abbreviations are used in this manuscript:

HMGCLD	3-hydroxy-3-methylglutaryl-CoA lyase deficiency
HMGCL	3-hydroxy-3-methylglutaryl-CoA lyase gene
HMG-CoA	3-hydroxy-3-methylglutaryl coenzyme A
NPO	Nil per os
PICU	Pediatric Intensive Care Unit
ASA	American Society of Anesthesiologists
IV	Intravenous
ABG(s)	Arterial Blood Gas(es)
PaO_2_	Partial pressure of oxygen in arterial blood
FiO_2_	Fraction of inspired oxygen
PaCO_2_	Partial pressure of carbon dioxide in arterial blood
BE	Base excess
HCO_3_^−^	Bicarbonate
NaCl	Sodium chloride
KCl	Potassium chloride
MgSO_4_	Magnesium sulfate
MAC	Minimum alveolar concentration
PC	Pyruvate carboxylase
PDH	Pyruvate dehydrogenase
LDH	Lactate dehydrogenase
ALT	Alanine aminotransferase
PEPCK	Phosphoenolpyruvate carboxykinase
G6Pase	Glucose-6-phosphatase
G3PDH	Glyceraldehyde-3-phosphate dehydrogenase
NADH	Nicotinamide adenine dinucleotide (reduced form)
FADH_2_	Flavin adenine dinucleotide (reduced form)
